# Interpenetrating network design of bioactive hydrogel coatings with enhanced damage resistance†

**DOI:** 10.1039/d2tb02825e

**Published:** 2023-02-20

**Authors:** Megan Wancura, Abbey Nkansah, Malgorzata Chwatko, Andrew Robinson, Ashauntee Fairley, Elizabeth Cosgriff-Hernandez

**Affiliations:** a Department of Chemistry, The University of Texas at Austin Austin TX 78712 USA; b Department of Biomedical Engineering, The University of Texas at Austin Austin TX 78712 USA cosgriff.hernandez@utexas.edu

## Abstract

Bioactive hydrogel coatings offer a promising route to introduce sustained thromboresistance to cardiovascular devices without compromising bulk mechanical properties. Poly(ethylene glycol)-based hydrogels provide antifouling properties to limit acute thromobosis and incorporation of adhesive ligands can be used to promote endothelialization. However, conventional PEG-based hydrogels at stiffnesses that promote cell attachment can be brittle and prone to damage in a surgical setting, limiting their utility in clinical applications. In this work, we developed a durable hydrogel coating using interpenetrating networks of polyether urethane diacrylamide (PEUDAm) and poly(*N*-acryloyl glycinamide) (pNAGA). First, diffusion-mediated redox initiation of PEUDAm was used to coat electrospun polyurethane fiber meshes with coating thickness controlled by the immersion time. The second network of pNAGA was then introduced to enhance damage resistance of the hydrogel coating. The durability, thromboresistance, and bioactivity of the resulting multilayer grafts were then assessed. The IPN hydrogel coatings displayed resistance to surgically-associated damage mechanisms and retained the anti-fouling nature of PEG-based hydrogels as indicated by reduced protein adsorption and platelet attachment. Moreover, incorporation of functionalized collagen into the IPN hydrogel coating conferred bioactivity that supported endothelial cell adhesion. Overall, this conformable and durable hydrogel coating provides an improved approach for cardiovascular device fabrication with targeted biological activity.

## Abstract 2

An interpenetrating network design was used to enhance the toughness of PEG-based hydrogel coatings to resist damage during surgical handling.

## Graphical abstract

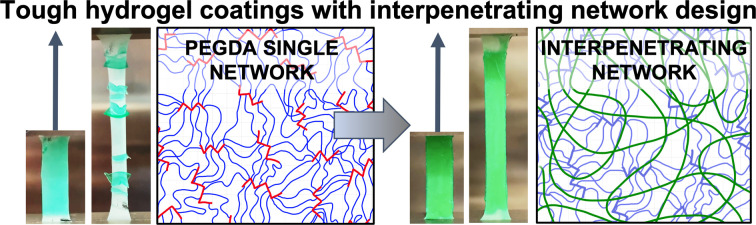

**10th Anniversary Statement**

The *Journal of Materials Chemistry B* has served to disseminate materials research advances in biology and medicine over the past decade. I was fortunate to join the editorial board as an Associate Editor in 2019. In addition to the quality and rigor of the scientific advances published by the journal, I have been continually impressed and inspired by the focus on how to better inspire, nurture, promote, and protect talent in the chemical sciences. The commitment for action on inclusion and diversity in publishing spans studies to examine bias in publishing practices, accessibility grants, trainee sponsorship opportunities, and numerous efforts to break down barriers. These efforts are constantly evolving and expanding with continued improvement of publishing and recognition practices. I have long believed that diverse teams of talented people are necessary to address global challenges in healthcare and it is a privilege to contribute to this mission and vision of the journal.

## Introduction

Blood-contacting devices such as small diameter vascular grafts and synthetic heart valves continue to fail due to thrombosis, adverse tissue reactions, and poor durability.^1–4^ Design of biomaterials that can combine thromboresistance and improved mechanical properties could expand treatment options and improve clinical outcomes. However, it continues to be challenging to achieve both biomechanical and biological criteria in a single material. Bioactive hydrogel coatings offer a promising route to introduce sustained thromboresistance to cardiovascular devices without compromising bulk mechanical properties.^5,6^ For example, our lab has developed a multilayered vascular graft with an electrospun mesh sleeve that provides arterial-matching mechanical properties and a polyethylene glycol (PEG)-based hydrogel coating with integrin-targeting bioactive properties to induce rapid endothelialization post-implantation for sustained thromboresistance.^7–9^ Prior research has demonstrated the acute thromboresistance of these grafts due to the antifouling nature of the hydrogel.^7,8,10–12^ Introduction of integrin-targeting proteins promoted endothelial cell adhesion, migration, and a hemostatic phenotype that can support sustained thromboresistance.^7,8,12–16^ Following these promising results, subsequent evaluation in porcine studies revealed surgical damage to the hydrogel coating of the vascular grafts. Suturing of the hydrogel caused particulates to be dislodged, resulting in embolism, and stretching damage exposed the underlying mesh and caused thrombosis.^8^ Despite a long history of investigation as antifouling coatings, there is a dearth of PEG-based hydrogel coatings in clinical use. We posit that it is the failure mechanisms such as the surgical damage described above that limit their utility in clinical applications. Hydrogels fabricated from PEG-diacrylate macromers less than 10 kDa are relatively brittle, with ultimate elongation <100%. Increasing macromer molecular weight can be used to increase the ultimate elongation; however, this also results in increased swelling and reduced stiffness that may not be desirable for biological interactions.^16^ New hydrogel chemistries are needed that can improve the durability of these antifouling coatings to enable use in cardiovascular devices.

Researchers have utilized network design to address the poor mechanical properties of conventional hydrogels that lead to failure with a focus on increasing extensibility and fracture-resistance.^17–19^ Approaches fall into four main categories: secondary interactions (ionic,^20–22^ coordination complexes,^23–25^ hydrogen bonds,^26–40^ hydrophobic domains^41–44^), homogenization,^45,46^ high functionality crosslinks,^47–52^ and interpenetrating networks (IPN)^53–61^ including double networks.^62–67^ Much of this research has focused on expanding available hydrogel chemistries^68–73^ and development of fundamental soft material mechanics.^74–79^ Recently, researchers have begun to focus on biomedical applications of these materials.^80–85^ Cardiovascular device design requires additional consideration of factors such as antifouling properties, hemocompatibility, biostability, and favorable cell–material interactions.^86^ We previously demonstrated that introduction of sacrificial hydrogen bonds could be used to reduce suture damage and resultant particle generation. Copolymerization of *N*-vinyl pyrrolidone during single network hydrogel fabrication increased fracture energy and correlated with suture damage resistance.^8^ This composition maintained requisite material properties for cardiovascular application including thromboresistance and endothelial cell adhesion and spreading. Although this eliminated one mode of hydrogel failure, it did not address hydrogel fracture due to stretching and surgical handling of the graft during implantation.

In contrast to suture damage, which correlated to fracture energy, our initial testing revealed that surgical damage was more strongly predicted by poor extensibility. The relatively high crosslink density of lower molecular weight acrylated PEG macromers (2–10 kDa) results in a brittle hydrogel with elongations less than 100%.^5^ Decreasing the crosslink density of these networks *via* increased macromer molecular weight (20–30 kDa) results in increased extensibility but also reduced modulus and increased swelling.^87^ Substrate stiffness of these networks is an important consideration given the established link to endothelial cell adhesion, spreading, and migration.^16,88–90^ IPN network approaches to hydrogel design provide opportunities to simultaneously increase hydrogel stiffness and network extensibility to a greater extent than is possible with single networks.^59^ Importantly, they retain high water contents necessary for antifouling surfaces.^91^ Yang and coworkers were able to achieve a two-fold increase in ultimate elongation and a four-fold increase in tensile modulus in functionalized PEG-based IPN hydrogels.^92^ The Hahn lab demonstrated collagen-PEG diacrylate IPN networks with moduli greater than either network alone that additionally limited platelet attachment and supported cell spreading.^53^ Thus, IPN networks have strong potential to improve hydrogel mechanical properties without sacrificing biomedical device design criteria.

Despite the advances in developing tough hydrogels, few of the new hydrogel chemistries can be applied as conformable coatings of medical devices or incorporate of biological molecules for targeted cell interactions.^93–95^ We previously reported a diffusion-mediated redox crosslinking methodology that facilitates the crosslinking of conformable PEG-based hydrogel coatings of tunable thickness with time and composition.^96^ In this work, we demonstrate an extension to this crosslinking methodology by using a sequential IPN approach to incorporate damage resistance and bioactivity to our first network while maintaining conformability.

**Fig. 1:**
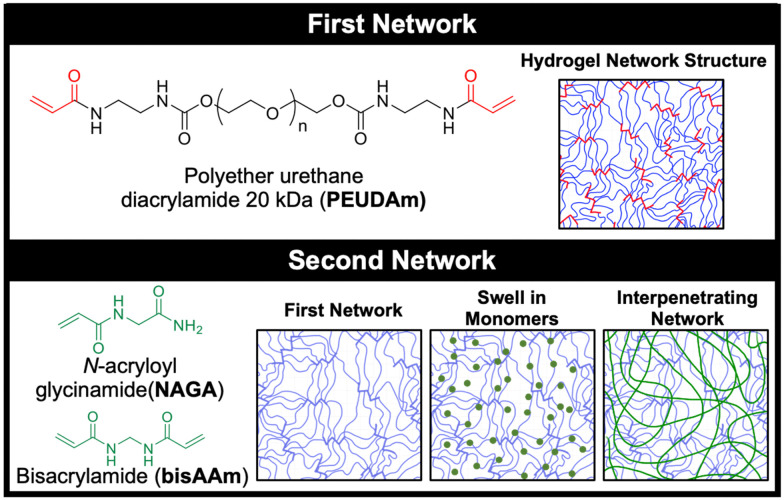
Hydrogel network structures. Single network PEUDAm hydrogels have a high functionality net point structure with hydrogen bonding groups near the crosslinking points. Covalent IPN hydrogels can be formed by swelling in the NAGA monomer and the bisAAm crosslinker and crosslinking within the first network. Hydrogen bonding secondary interactions within the second network and between the two networks enhances the mechanical properties of the IPN hydrogels.

In this study, we developed a durable hydrogel coating using interpenetrating networks of polyether urethane diacrylamide (PEUDAm) and poly(*N*-acryloyl glycinamide) (pNAGA, Fig. 1).^97^ First, mechanical properties of single network PEUDAm and IPN bulk hydrogels was used to identify a tough hydrogel composition. Conformable IPN coatings were then explored using a two-step approach combining redox diffusion mediated crosslinking and photopolymerization. Damage of multilayer composites under surgical handling (suturing, torquing, extension) and physiological loading was used to establish the durability of the IPN hydrogel coating. Finally, biological testing of the IPN hydrogel was used to confirm that the hydrogel retained its antifouling character and protein incorporation could be used to promote endothelial cell adhesion. Collectively, these studies establish the utility of a new hydrogel network design for application in cardiovascular devices. Hydrogel coatings are applicable to a wide range of biomedical devices and beyond, and this work will pave the way to future design of damage-resistant hydrogel coatings in many applications.

## Experimental

### Materials

Reagents were purchased from Sigma-Aldrich and used without further purification unless otherwise noted.

### Synthesis of polyether urethane diacrylamide (PEUDAm)

PEUDAm was synthesized in a three step process with intermediates of PEG with carbodiimidizole (CDI) and diamine endgroups. CDI (Alfa Aeser, 15 equivalent) was weighed in a dry, 500 mL round bottom flask and dissolved in minimal DCM (anhydrous, ∼10 mL). Polyethylene glycol (PEG 3.4, 10, or 20 kDa; 1 equivalent) was dissolved in anhydrous DCM (15 wt%) and degassed with nitrogen atmosphere. The PEG mixture was added dropwise to CDI over 2 hours at 30–35 °C, then stirred vigorously overnight at room temperature. The product was washed with deionized water three times in a separatory flask, dried with sodium sulfate, and collected *via* vacuum filtration. Solvent was removed under vacuum with a final drying under vacuum at 65 °C for 1 hour. H^1^ NMR (CDCl_3_): PEG-CDI: *δ* = 8.12 (s, 2H, –N–C*H*–N–), 7.40 (s, 2H, –N–C*H*=CH), 7.02 (s, 2H, –CH=C*H*–N), 4.51 (m, 4H, –O–C*H*_*2*_–CH_2_–), 3.70 (m, 1720H, –O–C*H*_2_–CH_2_–).

The resulting PEG-CDI product (1 equivalent) was then dissolved in anhydrous DCM (15 wt%) in a dry round bottom flask. Ethylenediamine (15 equiv.) was added to a dry 500 mL round bottom flask under nitrogen atmosphere and diluted with minimal DCM (5–10 mL). PEG-CDI was added dropwise to ethylenediamine at room temperature over 3 hours (1 drop per 1–3 seconds) under nitrogen atmosphere, and the mixture was stirred overnight. The product was washed with deionized water three times in a separatory flask, dried with sodium sulfate, and collected *via* vacuum filtration. The reaction was precipitated in 10× volume of ice-cold ether and collected *via* vacuum filtration. The product was dried under vacuum at 65 °C for 3 h. H^1^ NMR (CDCl_3_): *δ* = 4.18 (t, 4H, –O–C*H*_*2*_–CH_2_–), 3.60 (m, 1720H, –O–C*H*_2_–CH_2_–), 3.19 (m, 4H, –CH_2_–C*H*_2_–NH), 2.78 (m, 4H, –NH_2_–C*H*_2_–CH_2_–), 3.70 (m, 1720H, –O–C*H*_2_–CH_2_–).

In the final functionalization step, the PEG-diamine product (1 equivalent) was weighed in a dried 500 mL 3-neck round bottom flask and dissolved in anhydrous DCM at 15 wt% under nitrogen. Triethylamine (2 equiv.) was added and then acryloyl chloride diluted in DCM (∼4 mL) was added dropwise (1 drop per 4–5 s). The reaction was allowed to proceed for 24 hour at room temperature, then an additional 2 equiv. of acryloyl chloride was added dropwise. The reaction was continued for another 24 hour at room temperature, then quenched with potassium carbonate (2 M, 8 equiv.) The product was washed with water two times, dried with sodium sulfate, and collected *via* vacuum filtration. Precipitation was run in ice-cold diethyl ether and collected *via* vacuum filtration. The final product was dried at atmospheric pressure overnight, then briefly under vacuum. Polymers with percentage conversions of hydroxyl to acrylamide end groups over 80% were used in this work. H^1^ NMR (CDCl_3_): *δ* = 6.94 (broad s, 2H, –C–N*H*–CH_2_–), 6.28 (dd, 2H, *H*_2_C=CH–C–), 6.17 (m, 2H, H_2_C=C*H*–C–), 5.85 (broad s, 2H, –C–N*H*–CH_2_–), 5.61 (dd, 2H, *H*_2_C=CH–C–), 4.20 (m, 4H, –H_2_C–C*H*_2_–O–), 3.65 (m, 1720H, –O–C*H*_2_–CH_2_–), 3.34 (m, 4H, –CH_2_–C*H*_2_–NH–).

### Bulk hydrogel fabrication and characterization

PEUDAm hydrogel solutions were prepared by dissolving PEUDAm (3.4, 10, or 20 kDa) in deionized water at a concentration of 10, 15, or 20 wt/vol%. Irgacure 2959 (10 wt/vol% solution in 70 vol% ethanol) was added at a final concentration of 0.1 wt/vol%. NAGA hydrogel solutions were prepared by dissolving *N*-acryloyl glycinamide (NAGA; BDL Pharma) in deionized water at a concentration of 20 wt/vol%. Irgacure 2959 was added at a final concentration of 0.1 wt/vol% and bisacrylamide was added at 0.1 mol% relative to NAGA. Bulk hydrogels were fabricated by placing precursor solutions between 1.5 mm spaced glass plates and curing on a UV transilluminator (UVP, 25 watt, 365 nm). PEUDAm hydrogels were cured for 6 minutes on both sides. pNAGA hydrogels were cured for 20 minutes on both sides.

IPN hydrogels were fabricated *via* a sequential IPN method. First network hydrogels were prepared identically as above with 10 wt/vol% PEUDAm 20 kDa macromer solutions. Hydrogels were swollen to remove residual macromer, then dried at ambient pressure overnight. Dried hydrogels were placed in a vial with a solution of NAGA (10, 15, or 20 wt/vol%), bis-acrylamide (0.1 mol% relative to NAGA), and Irgacure 2959 (2 wt/vol%). Soaking solutions were rotated overnight at room temperature and protected from light. IPNs were formed by placing monomer-swollen hydrogels between glass spacer plates set to the swollen thickness and curing on a UV transilluminator for 20 minutes on both sides.

Swelling ratios and sol fractions were determined for each hydrogel composition (*n* = 12). Circular hydrogel specimens (*D* = 8 mm) of single networks and IPNs were dried under vacuum immediately after fabrication and weighed (*W*_i_). Specimens were weighed after swelling in deionized water for 24 h (*W*_s_), and then weighed again after drying under vacuum for 24 h to assess dry polymer mass (*W*_d_). The sol fraction was calculated as (*W*_i_ − *W*_d_)/*W*_i_. The equilibrium volumetric swelling ratio, *Q*, was calculated from the equilibrium mass swelling ratio: *W*_s_/*W*_d_.

### Hydrogel mechanical testing

Mechanical testing was performed to determine the effect of compositional variables on tensile and fracture properties of the hydrogels. Hydrogels of each composition were fabricated as described above and tested at equilibrium swelling conditions. Tensile testing was performed on dog-bone shaped hydrogels of all compositions (SN: 10 wt/vol% PEUDAm 3.4, 10, and 20 kDa; 15 and 20 wt/vol% PEUDAm 20 kDa; 20 wt/vol% pNAGA; IPNs: (1) 10 wt/vol% PEUDAm 20 kDa, (2) 10, 15, or 20 wt/vol% pNAGA + 0.1 mol% bisAAm). Bulk hydrogels were cut to dog-bone shapes using a custom 3D printed device as described by Nelson and coworkers.^98^ Thickness ranged from 0.12–0.17 mm due to variations in hydrogel swelling among molecular weights. Testing was performed using a Dynamic Mechanical Analyzer (TA Instruments). Hydrogel specimens (*n* = 12) were strained to fracture at a rate of 0.1 mm s^−1^. Only the specimens that fractured in the gauge length and not at the grips were utilized for analysis. Specimen ultimate elongation, ultimate tensile strength, and modulus between 5–15% strain were then calculated.

Fracture energy characterization was performed on a smaller selection of hydrogels (SN: 10 wt% PEUDAm 20 kDa; pNAGA, 20 wt%; IPNs: (1) 10 wt% PEUDAm 20 kDa, (2) 20 wt% pNAGA + 0.1 mol% bisAAm). Fracture energy was characterized using a single edge notch mechanical test. Hydrogel specimens were fabricated at a geometry of 30 mm long, 10 mm wide, and 1.2–1.5 mm thick. Single network (10 wt/vol% PEUDAm 20 kDa, 20 wt/vol% pNAGA + 0.1 mol% bisAAm) and IPN hydrogels ((1) 10 wt/vol% PEUDAm 20 kDa, (2) 20 wt/vol% pNAGA + 0.1 mol% bisAAm) were characterized. A 5 mm long notch was cut halfway along the length of the gel with a razorblade. Hydrogels were loaded into tensile grips (RSAIII DMA) using sandpaper to prevent slipping. Hydrogels were extended in tension at 1 mm s^−1^ until the crack had propagated to failure. Fracture toughness was determined as the area of force to fracture (*F*) over the length of fracture (*d*). Fracture energy was determined by dividing fracture toughness by the thickness (*t*) of the hydrogel times the length (*L*_bulk_) of the gel to be fractured, 
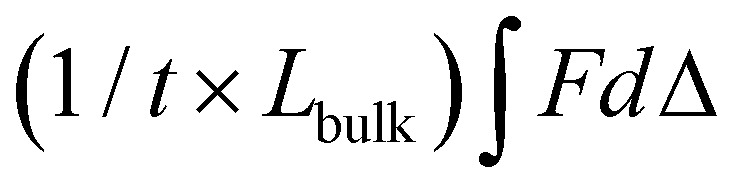
.

### Fabrication of electrospun meshes

To assess composites with hydrogel coatings, electrospun fiber meshes were fabricated in flat sheets and tubes. For flat sheets, an electrospinning solution was prepared from Bionate® 80A (DSM Biomedical Inc., Berkeley, CA, USA) by dissolving the polymer in 70 : 30 dimethylacetamide : tetrahydrofuran at 14 wt%. The solution was pumped at a rate of 0.5 mL h^−1^ through a 20-gauge blunt needle. Fibers were collected on a rotating mandrel at a working distance of 50 cm and a rotational speed of 50 rpm. The needle tip was charged to a range of +11.5–15.5 kV using a high voltage source to maintain Taylor cone stability with the mandrel charged at −5 kV. A spinning time of 3.5–4 hours produced meshes in a thickness range of (0.13–0.18 mm, *n* = 15). Relative humidity was controlled between 40–50% with a temperature range of 21.0–26.5 °C. Upon completion of the spin, meshes were annealed on the mandrel at 70 °C overnight and then removed by cutting down the center of the mandrel. Tubular grafts were fabricated from Bionate® Segmented Polyurethane (DSM Biomedical Inc., Berkeley, CA, USA) that was dissolved to 24 wt% in dimethylacetamide. Electrospun polyurethane mesh tubes were fabricated by pumping polymer solutions at a rate of 0.5 mL h^−1^ through a 20-gauge blunt needle with fibers collected on a 5 mm rotating rod (500 rpm) positioned 50 cm from the needle tip. Prior to spinning, the rod was dip coated in a 5% 35 kDa PEG in dichloromethane solution to facilitate graft removal. The 5 mm mandrel was charged at −5 kV and the needle tip at +17–20 kV using a high voltage source. Spinning was run for one hour until the desired thickness was achieved (∼0.15 mm). Tubular grafts were spun at ambient conditions (relative humidity 45–50%, temperature 21–22 °C). Following spinning, the rods were submerged in water for a minimum of one hour to dissolve the PEG layer and enable graft removal. Three sections were taken from each mesh with each section imaged in five locations under scanning electron microscopy for a total of 15 images per mesh (Fig. S1, ESI†).

### Composite fabrication and characterization

Photopolymerized hydrogel coatings of electrospun meshes was performed as previously described.^8^ Briefly, electrospun mesh sleeves were taken through a graded ethanol/water soak (70 vol%, 50 vol%, 30 vol%, and 0 vol%, 15 min each) to ensure aqueous solution penetration. Meshes were soaked in a precursor solution of 10 wt/vol% PEGDA 3.4 kDa. Then, the polymer-soaked substrates were placed in a cylindrical mold with an inner glass mandrel (3 mm OD). Precursor solution was added to the mold and crosslinked with UV light for 12 min in a custom-built UV box.

Diffusion-mediated redox initiated crosslinking of hydrogel coating of electrospun polyurethane meshes was performed as previously described with minor modifications.^96^ Briefly, mesh substrates (0.12–0.19 mm thick) were passed through a graded ethanol ramp (70 vol%, 50 vol%, 30 vol%, and 0 vol% ethanol in water, 15 minutes each) prior to use to ensure adequate wetting of the substrate. Meshes were coated in a solution of iron gluconate (IG, 3 wt/vol% [Fe^2+^] as determined with the Ferrozine Assay^99^) *via* adsorption by soaking ramped meshes in IG for 15 minutes. After soaking, meshes were briefly dipped in methanol to facilitate uniform drying, then dried under compressed air for one minute. After drying, meshes were immediately transferred to 3D printed clamps and immersed in aqueous solutions of 10 wt/vol% PEUDAm 20 kDa and ammonium persulfate (APS, 0.05 wt/vol%). For thickness studies, IG coated meshes (5 × 10 mm, *n* = 12) were immersed for 10, 20, or 30 seconds in a 96 well plate. For all other studies, IG coated meshes (20 × 20 mm, *n* = 1 to yield 4, 8 mm punches) were immersed for 10 or 20 seconds in a custom 3D printed well plate. After fabrication, composites were allowed to continue crosslinking until dry (2 hours to overnight). All composites were washed in deionized water with three exchanges of water at 10 min, 15 min, and overnight to remove the soluble macromer fraction.

IPN hydrogel coatings were fabricated in a sequential manner similar to bulk gels. Dry, hydrogel coated meshes fabricated as described above were soaked in a solution of 20 wt/vol% pNAGA, 0.1 mol% bisAAm, and 2 wt/vol% Irgacure 2959 overnight protected from light at 0 °C. IPN coatings were placed on glass slides without molding and were cured *via* photopolymerization on a UV transilluminator for 12 minutes on each side. IPN coated specimens were prepared for use in the following assays by washing in deionized water with three exchanges to remove the sol fraction.

Thickness of hydrogel coatings was measured as a function of time (10, 20, and 30 s, *n* = 12) and after addition of the second pNAGA network. First network coating thickness was measured after overnight swelling and trimming of edges using a force-normalized caliper (Mitutoyo). Final thicknesses of the swollen IPN coatings were determined after crosslinking and overnight swelling. Thicknesses immediately after crosslinking were difficult to characterize due to dimensional changes during crosslinking caused by evaporation.

### Stretching damage

Delamination of the hydrogel coating after uniaxial stretching was assessed as a measure of coating durability. Hydrogel composites were prepared from photopolymerized PEGDA 3.4 kDa or large (20 × 20 mm) IPN composites at swollen thicknesses of 0.3 and 0.8 mm (*n* = 6). Samples were cut to 15 × 5 mm. Hydrogels were dyed with food coloring for better visualization of failure. The composite materials were placed between the grips of a Dynamic Mechanical Analyzer (TA Instruments) and strained to 100% at a rate of 1 mm s^−1^. Composite stretching was video recorded. The point at which visible damage to the hydrogel coating occurred was noted and strain at failure was determined at this point. Strain was determined by measuring the length of the hydrogel coating region before and at the end of testing to minimize the effect of the electrospun mesh substrate yielding.

### Suture damage

Suture-induced damage was determined as described previously by Post *et al.*^8^ Briefly, a 7-0 suture (Ethicon) needle and thread were passed through 3.4 kDa and IPN hydrogel coatings (*n* = 9). The number of particulates dislodged was visualized and counted under a stereoscope (Olympus SZ61).

### Twisting damage

Twisting-induced damage of hydrogel composites was characterized for IPN hydrogel coatings as compared to 3.4 kDa PEGDA photopolymerized hydrogel coatings (*n* = 3). Tubular composites (5 mm inner diameter, 4 cm long, 0.2 mm inner hydrogel thickness) were gripped in the center region tightly with forceps and twisted 180°. Composites were cut open longitudinally and damage to the inner hydrogel coating was visualized under a stereoscope (Olympus SZ61).

### Physiological conditioning

Resistance to delamination under pulsatile flow was characterized for IPN hydrogel coating of tubular meshes. Tubular composites (5 mm inner diameter, 4 cm long, 0.2 mm inner hydrogel thickness) were sterilized *via* ethylene oxide (*n* = 3). A physiological flow setup was developed with a Masterflex L/S variable speed peristaltic pump (Cole-Parmer) fitted with a Masterflex L/S pump head to provide pulsatile flow and induce pressure at intraluminal pressure (120/80 mm Hg). Pressure was maintained with an external pump (kd Scientific) set to a flow rate of 1.6 mL h^−1^ and monitored (SSI Technologies, Inc. MediaGauge). Tubular composites were mounted to the testing chamber and ends were secured to Teflon adapters. The flow loop was filled with a 45% glycerin solution with a dynamic viscosity of 3.5 mPa s at 37 °C,^100^ similar to blood in the coronary artery.^101^ Grafts were placed in a 37 °C water bath and tested at a flow rate of 2 mL s^−1^ for a period of 1 week. Grafts were removed from the flow loop, the distal and luminal ends were cut off, and the remainder of the construct was cut open. Delamination at the graft edges and along the center was visually assessed.

### Protein adsorption

Protein adsorption to hydrogel coatings (3.4 kDa, IPN), uncoated mesh samples, and ePTFE vascular grafts (DotMed) was determined as described by Swartzlander *et al.* with minor modifications.^102^ Uncoated mesh and ePTFE samples were prepared with a graded ethanol ramp (70, 50, 30, 0 vol% ethanol/water). ePTFE grafts were additionally soaked in degassed PBS to promote wetting of the superhydrophobic substrates.^103^ All samples were cut to 8 mm and soaked in FBS overnight at 37 °C. FBS was removed and samples were washed once with PBS. Samples were moved to a solution of 50 mM ammonium bicarbonate and sonicated for 15 minutes at 40 °C. Solutions were snap frozen in liquid nitrogen and lyophilized. Total protein mass was quantified using the bicinchoninic acid (BCA) assay kit (BioVision) per manufacturer's instructions (*n* = 12). Absorbance measurements were taken (Tecan Infinite M Nano+) and results analyzed *via* a standard curve.

### Platelet attachment

Platelet attachment was characterized as described previously as an initial measure of thromboresistance.^8^ IPN and PEGDA 3.4 kDa hydrogel composites, uncoated mesh samples, and ePTFE vascular grafts (DotMed) were prepared as described for protein adsorption. Samples (8 mm, *n* = 12) were soaked in FBS overnight at 37 °C, washed once with PBS to remove non-adhered protein, and placed in a 48 well plate. Platelets were isolated from human whole blood drawn from a volunteer and mixed with heparin *via* inversion. Informed written consent was obtained from the volunteer as described in the approved IRB protocol STUDY00002311. Whole blood (8 mL) was centrifuged at 990 rpm for 15 minutes to isolate the protein rich plasma (PRP) layer. The PRP layer was removed, prostacyclin added at 10 μL mL^−1^, and centrifuged again at 1500 rpm for 10 min to form a platelet pellet. The pellet was resuspended in CGS buffer for washing and centrifuged again at 1500 rpm for 10 min. The platelets were then resuspended in Tyrode's buffer at half the original volume of PRP. Sudan B Black solution (5% in 70% ethanol) was added to the platelet solution at a 1 : 10 ratio for 30 minutes at room temperature. The stained platelets were then washed with PBS 3 times by resuspending the pellet in PBS then centrifuging at 1500 rpm for 8 min. Platelets were counted and resuspended at a concentration of 10 × 10^6^ platelets per mL in sterile PBS. The platelet suspension (500 μL) was added in each test well, and platelets were allowed to adhere to substrate for 30 min at 37 °C on a shaking incubator. Samples were transferred to new wells and washed twice with PBS, then carefully placed into a new 48 well plate. Bound cells were lysed with 150 μL DMSO for 15 min at room temperature, then 150 μL PBS was added. Solutions were moved in triplicate to a 96 well plate and absorbance was read on a spectrophotometer (Tecan Infinite M Nano+).

### Endothelial cell adhesion

Bioactivity was conferred to the hydrogels *via* covalent incorporation of collagen functionalized with acrylamide-PEG-isocyanate linker as described previously (functionalization of ∼10% of the available lysines).^104^ For bulk hydrogels, collagen was added directly to the polymer solution at 6 mg mL^−1^. For IPN hydrogel coatings, an aqueous solution of collagen (6 mg mL^−1^, 100 μL) was added on top of the NAGA-swollen composites prior to photocuring. Circular specimens (*D* = 8 mm) were punched from hydrogel slabs or 20 × 20 mm coatings with and without collagen and placed into a 48 well plate. Human umbilical vein endothelial cells (HUVECs) were cultured in EGM-2 cell media (Lonza), harvested for use at passage P4-P6, and seeded at 12 000 cells per well. Cells were allowed to attach for 3 hours, then washed twice with warm 10 mM phosphate buffered saline (PBS). Specimens were fixed with 3.7% glutaraldehyde and stained with rhodamine phalloidin (actin/cytoskeleton) and SYBR green (DNA/nucleus). Samples were imaged with a fluorescence microscope (Nikon Eclipse TS100) and cell adhesion was quantified (*n* = 12).

### Statistical analysis

The data for all measurements are displayed as mean ± standard deviation. An analysis of variation (ANOVA) comparison utilizing Tukey's multiple comparison test was used to analyze the significance of data among multiple compositions. All tests were carried out at a 95% confidence interval (*p* < 0.05).

## Results and discussion

In this study, a bioactive and thromboresistant hydrogel coating that successfully resists surgical damage is described. An IPN network design was chosen to achieve simultaneous high water content, stiffnesses appropriate for endothelial cell adhesion and spreading, and damage resistance. To this end, a new PEG-based macromer with enhanced biostability was synthesized, PEUDAm. The first study of this work aimed to characterize baseline properties of PEUDAm hydrogel networks including the effects of molecular weight and macromer concentration. IPN networks were then investigated with a first network of PEUDAm 20 kDa and a second network of *N*-acryloyl glycinamide (NAGA), a modified amino acid monomer with bidentate hydrogen bonding, with a bisacrylamide crosslinker (Fig. 1). Tensile and fracture properties were characterized for IPN networks to identify appropriate properties for medical device coatings. Namely, modulus values similar to PEGDA 3.4 kDa for endothelial cell adhesion and high ultimate elongations and mechanical energy dissipation for damage resistance were targeted. We then adapted our redox-initiated coating method to apply conformable IPN hydrogel coatings to electrospun meshes. Finally, we characterized the ability of these coatings to resist surgical damage while maintaining appropriate biological properties.

### Single network analysis of PEUDAm hydrogel

Tensile properties PEUDAm single networks were determined at multiple molecular weights and concentrations (Fig. S2, ESI†). Increased PEUDAm macromer molecular weight significantly increased ultimate elongation and decreased modulus (*p* < 0.0001, *n* = 12) but did not significantly affect ultimate tensile strength. Increased macromer concentration for 20 kDa PEUDAm networks resulted in significantly decreased ultimate elongations, increased modulus, and increased ultimate tensile strengths (*p* < 0.005, *n* = 12). The effect of molecular weight and concentration on crosslinking density are well established in the literature for PEG diacrylate (PEGDA) networks at molecular weights up to 10 kDa.^5,105^ For end-functionalized macromers, increases in molecular weight correspond to decreased crosslinking density as indicated by decreased modulus. This trend is demonstrated for 10 wt% PEUDAm networks from 3.4 to 20 kDa. Increased modulus with macromer concentration (10 *vs.* 20 wt%) was also observed for PEUDAm 20 kDa networks.^106^ These results indicate PEUDAm networks follow literature precedence for end-functionalized macromer hydrogels similar to PEGDA networks (Fig. S3 and Table S1, ESI†).

### Bulk IPN hydrogel mechanical testing to identify a durable formulation

**Fig. 2:**
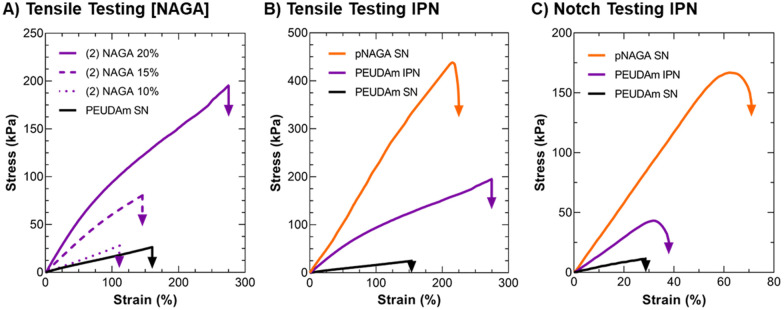
Tensile and fracture properties of IPN hydrogels in comparison to component networks. (A) Effect of NAGA concentration on IPN uniaxial tensile properties. (B) Comparison of IPN uniaxial tensile properties to component single networks. (C) Comparison of IPN single notch tensile properties to component single networks. Unless otherwise specified, concentrations of IPN are 10 wt% PEUDAm 20 kDa in the first network and 20 wt% pNAGA with 0.1 mol% bisAAm in the second network. Representative curves are presented, *n* = 12.

After characterizing PEUDAm single network properties, tensile properties of IPN bulk hydrogels were assessed (Fig. 2). First, the effect of NAGA concentration on IPN properties was investigated. Increased NAGA concentration from 10 to 20 wt% corresponded to significantly increased ultimate elongations, ultimate tensile strengths, and moduli (*p* < 0.0001, *n* = 12, Fig. 2(A)). IPNs fabricated from 20 wt% NAGA had a much stronger impact on mechanical properties than 10 or 15 wt% NAGA IPNs (Fig. S4, ESI†). Second network incorporation at 20 wt% NAGA resulted in increased ultimate elongations (SN: 170 ± 36% *vs.* IPN: 280 ± 76%, *p* = 0.0003; *n* = 12), tensile modulus (SN: 20.0 ± 2.02 *vs.* IPN: 118 ± 24.8 kPa; *p* < 0.0001, *n* = 12), and ultimate tensile strength (SN: 27 ± 7.7 *vs.* IPN: 240 ± 71 kPa, *p* < 0.0001; *n* = 12), Fig. 2(B). For all properties, 20 wt% pNAGA hydrogels had the highest results (Table S2, ESI†). PEUDAm IPNs also displayed increased modulus, tensile strength, and ultimate elongation as compared to PEGDA IPN hydrogels, Fig. S5 (ESI†).

Incorporation of the pNAGA secondary network into PEUDAm network increased crosslinking density as evidenced by the increase in modulus and decreased swelling ratio. The second network also improved the extensibility and ultimate tensile strength of the IPN hydrogel. IPNs conventionally show synergistic effects between the two networks. In this study, the IPN showed properties intermediate to the PEUDAm and pNAGA single networks. The high strength of pNAGA hydrogels is due to their extensive and highly stable hydrogen bonding interactions.^97^ Dai and coworkers proposed pNAGA hydrogels to be crosslinked *via* hydrogen bonding microdomains that are strong enough to suppress hydration within hydrogels. The formation of the pNAGA as an interpenetrating network where it is entangled with PEG chains likely suppresses the extensive formation of these hydrogen bonding microdomains observed in the single pNAGA network hydrogels. We hypothesize that there is hydrogen bonding between the pNAGA network and PEUDAm network. The similarity of the mechanical properties and swelling between PEGDA and PEUDAm single networks indicated a low degree of hydrogen bonding between the urethane moieties in the PEUDAm network. However, PEUDAm-based IPNs displayed enhanced properties as compared to PEGDA-based IPNs, which supports hydrogen bonding between the pNAGA network and PEUDAm networks, Fig. S5 (ESI†).

The impact of the IPN structure on mechanical energy dissipation was explored *via* fracture energy characterization. Single edge notch tests were conducted on PEUDAm IPN networks relative to the component single networks (Fig. 2(C)). For PEUDAm, the maximum force at fracture was 0.07 ± 0.04 N, the maximum elongation at break was 26 ± 6%, and the fracture energy was 0.03 ± 0.02 J mm^−2^. For the IPN, the maximum force at fracture increased to 0.34 ± 0.09 N, and the maximum elongation at break increased to 34 ± 5.0% (*p* < 0.0001 for maximum force and *p* < 0.05 for fracture elongation, *n* = 12). Fracture energy was not significantly increased (0.22 ± 0.08 J mm^−2^ as compared to 0.03 ± 0.02 J mm^−2^ for the SN, *p* = 0.0878). Fracture properties of the pNAGA SN were significantly greater than IPN for all conditions, achieving fracture energy values of 1.63 ± 0.38 J mm^−2^ (*p* < 0.0001, *n* =12). As with tensile property characterization, fracture energy characterization also displayed IPN with properties intermediary to the two component networks. In pNAGA networks, sacrificial hydrogen bonding interactions are broken to dissipate fracture energy.^66^ Sun and coworkers reported a mechanism for mechanical energy dissipation in IPN networks, whereby a polyacrylamide gel was crosslinked with an alginate gel.^20^ As mechanical force was applied to a fracture in the network, the brittle polyacrylamide bonds fracture and mechanical energy from the fracture is dissipated to disrupt hydrogen bonds in the alginate network as opposed to propagating through the polyacrylamide network. In this work, a similar mechanism is likely employed. Again, disruption of the hydrogen bonding microdomain decreases the ability of the pNAGA hydrogel to dissipate fracture energy. Regardless, the IPN demonstrated substantial strengthening as compared to the PEUDAm single network. Collectively, these studies identified a candidate IPN hydrogel with target mechanical properties, stiffness to support endothelial cell attachment, high extensibilities, and mechanical energy dissipation.

### Conformal IPN hydrogel coatings with diffusion-mediated redox crosslinking

**Fig. 3:**
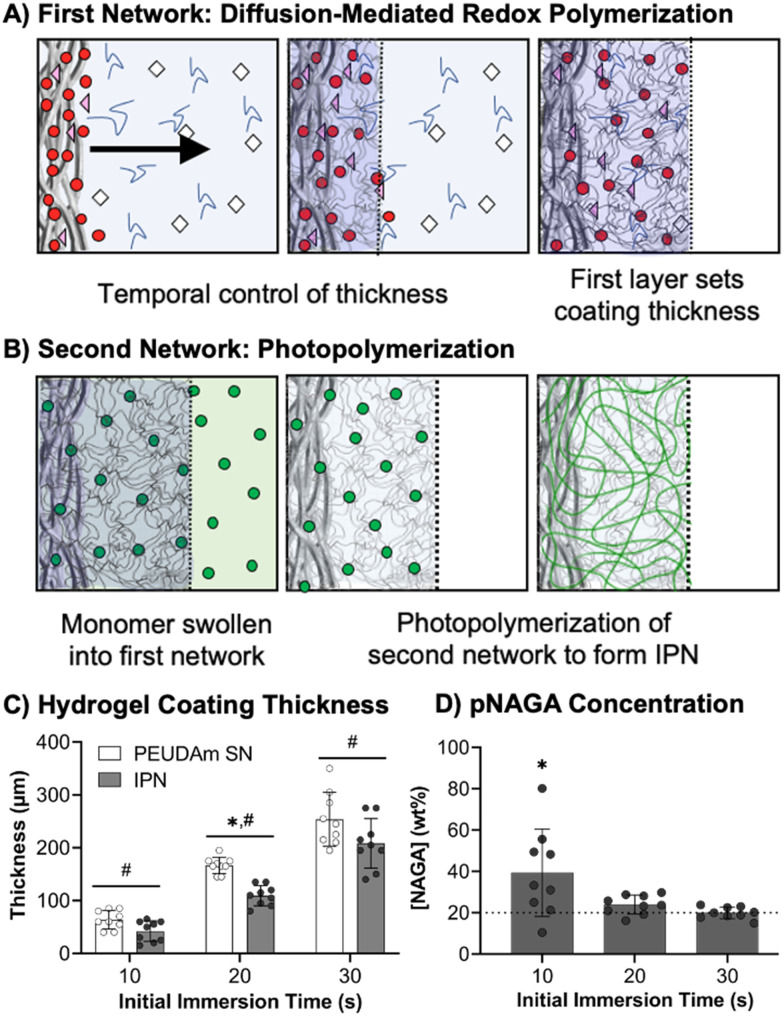
Schematic of IPN hydrogel coating process. (A) Diffusion-mediated redox initiated crosslinking of the PEUDAm first network sets the thickness of the hydrogel coating layer. (B) NAGA, bisAAm, and photoinitiators are then swollen into the first network and cured *via* photoinitiation. (C) Effect of immersion time on coating thickness of the first network and the second network at equilibrium state (*n* = 9). (D) Effect of immersion time on second network concentration (*n* = 9).

After characterizing bulk properties to identify a tough hydrogel composition, we then developed the methodology to apply this IPN hydrogel as a device coating using electrospun meshes as a model substrate. Previously, our lab reported a conformable crosslinking methodology based on a diffusion-mediated redox crosslinking mechanism.^96^ This method successfully forms PEGDA hydrogel coatings at molecular weights up to 20 kDa with thickness tunable by time and initiator concentration. We hypothesized that applying the IPN approach to these materials would give robust and damage resistant coatings. In these studies, first network coatings were fabricated with 10 wt% PEUDAm 20 kDa with the redox diffusion-mediated method for 10, 20, or 30 s immersion times, Fig. 3(A). Then, NAGA solutions were soaked into the networks overnight and photocured to set the second network, Fig. 3(B). The thickness of the coating was characterized after equilibrium swelling and used as a measure of the swelling ratio of the IPN, Fig. 3(C).

Hydrogel thicknesses for the first network increased with immersion time from 56 ± 18 μm at 10 s to 258 ± 37 μm at 30 s (*n* = 9). Thickness differences at all time points were significant (*p* < 0.001, *n* = 9). Hydrogel thickness growth with time was maintained in the final IPN network with thicknesses ranging from 50 ± 25 μm at 10 s to 176 ± 30 μm at 30 s (*n* = 9). Thickness differences at all time points remained significant (*p* < 0.0001, *n* = 9). Differences in thickness between the first and second networks were significant at 20 and 30 s (*p* < 0.0001, *n* = 9). The effective concentration of pNAGA in the second networks was characterized across first network immersion times as a measure of second network incorporation, Fig. 3(D). All immersion times resulted in concentrations close to 20 wt% with no significant differences (*n* = 9). For 20 and 30 s coatings, averages were 23 ± 5 wt% and 20 ± 3 wt%, respectively (*n* = 9).

The diffusion-mediated redox crosslinking method was successfully implemented with PEUDAm 20 kDa networks. Growth kinetics enabled a 5-fold increase in thickness over a 20 s-time difference in immersion. IPNs were successfully fabricated for each immersion time, as confirmed with FTIR spectroscopy (Fig. S8, ESI†). It was hypothesized that decreases in thickness with IPN incorporation were a result of differences in swelling ratios between PEUDAm (29.0 ± 0.3) and pNAGA (3.4 ± 0.1) single networks with the IPN swelling ratio (6.1 ± 1.3) being similar to the pNAGA network, Fig. S9 (ESI†). Upon IPN fabrication, dimensional restrictions caused by increased crosslinking density of the pNAGA network prevent swelling to the same thickness as the first network alone. IPN coatings are predicted to have similar properties to the bulk hydrogels, as the effective incorporation matches concentrations of the bulk gels. However, further characterization of surface mechanical properties *via* nanoindentation is needed to validate this hypothesis. Importantly, IPN hydrogel coatings retain the conformability of the redox diffusion-mediated coating methodology. Thin hydrogel coatings are desirable for small diameter vascular grafts, as the luminal diameter is important and initially set by the substrate. Collectively, these studies demonstrate that the IPN coatings qualitatively improve network stiffness while maintaining conformability and tunable thickness control based on immersion time.

### Damage resistance of IPN hydrogel coatings

**Fig. 4:**
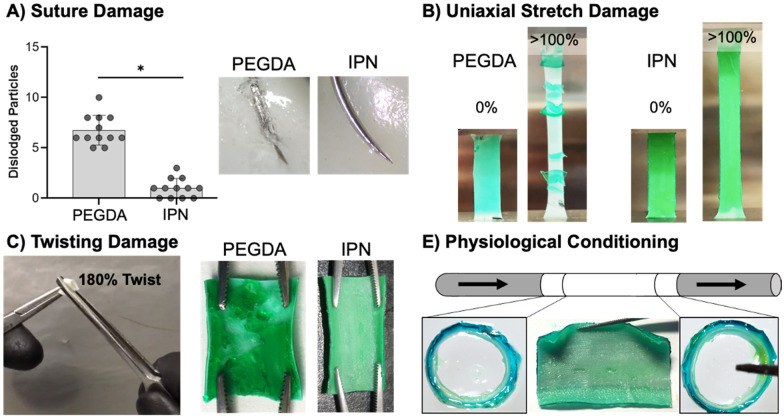
Characterization of damage resistance of IPN hydrogel coatings in comparison to photopolymerized PEGDA hydrogel coating. (A) Suture damage to hydrogel coating and particle generation during suturing (*n* = 12). (B) Damage of hydrogel coatings after uniaxial stretch (*n* = 6). (C) Damage of hydrogel coating on tubular constructs after gripping and twisting with forceps (*n* = 3). (D) Resistance of sterilized IPN hydrogel coated tubular grafts to delamination under pulsatile flow (*n* = 3). The * represents a significant difference at a 95% confidence interval.

This work set out to design a robust hydrogel coating capable of maintaining the bioactivity of previous small diameter vascular grafts achieved by our lab. After successfully fabricating IPN hydrogel coatings, we next moved on to characterize their resistance to various surgical-associated damage modes. Damage resistance of IPN hydrogel coatings was assessed relative to damage-prone PEGDA 3.4 kDa hydrogel coatings (Fig. 4). Namely, the hydrogel coating's resistance to suturing, stretching, twisting, and physiological conditioning was characterized.

Resistance to particulate generation during suturing is essential for material implantation. Passing a suture needle through hydrogel composites resulted in a significantly lower number of dislodged particles in IPN coatings as compared to 3.4 kDa coatings (*p* < 0.0001, *n* = 12, Fig. 4(A)). Previously, suture damage resistance was increased by incorporating *N*-vinyl pyrrolidone into PEGDA 3.4 kDa hydrogel networks.^8^ The incorporation of this monomer increased the fracture energy of the hydrogel network, and resistance to particulate generation was correlated to this mechanical property. Fracture properties of the IPN dwarf those of single network PEGDA hydrogels, and IPN coatings showed only a small amount of particulate generation (Fig. 4(A)). Fracture data for PEGDA networks was not presented in this work as only small differences between compositions were observed. IPN coating resistance to suturing damages can loosely be attributed to its ability to effectively dissipate mechanical energy. Hydrogel particulates can result in embolism if dislodged into the bloodstream. Future studies should characterize methods to eliminate particulate generation from the IPN coatings entirely, perhaps by exploring the impact of hydrogel thickness and improving suturing technique.

Stretching damage characterization was used as a qualitative measure of the cohesive strength of hydrogel coatings.^107^ Stretching of IPN coatings to measure the resistance of these networks to delamination and ability to deform under handling was an essential first pass in durability assessment (Fig. 4(B)). Stretching hydrogel composites to 100% strain resulted in failure of both thick and thin 3.4 kDa coatings but neither thick nor thin IPN coatings (*n* = 6). Thinner 3.4 kDa coatings were significantly more resistant to stretching failure than thicker coatings (*p* < 0.0001, *n* = 6, Fig. S10, ESI†). IPN coatings were able to strain to 100% without delamination or damage to the coatings (Fig. 4(B)). Thickness had a clear impact on the ability of the 3.4 kDa coatings’ ability to resist stretching damage with thick coatings leading to earlier failure. Both thick and thin IPN coatings were able to resist damage and delamination caused by stretching. This resistance is attributed to the increased extensibility and tensile strength of the IPN formulation.

Next, resistance of the hydrogel composites to twisting damage was determined to qualitatively assess resistance to damage under surgical forceps handling during implantation. A 180° twist was implemented as an extreme of what the materials may need to handle in a surgical suite. Hemostats were used to twist hydrogel coated vascular grafts 180° was while applying pressure (Fig. 4(C)). All 3.4 kDa coated grafts showed significant damage and exposure of the underlying substrate, whereas no IPN samples showed damage (*n* = 3, Fig. 4(C)). This resistance to damage is again attributed to high ultimate elongation as well as mechanical energy dissipation. Finally, the ability of IPN hydrogel coatings to resist delamination under physiological flow was assessed. Tubular graft composites cut to 4 mm were conditioned for one week at 37 °C under pulsatile flow set to adult physiologic conditions (*n* = 3, Fig. 4(E)). Upon removal, edges were sectioned, and the remainder of the sample was cut open to assess delamination. IPN composites did not show signs of delamination and cross sections at distal and ventral ends showed no damage (Fig. 4(E)). IPN hydrogel coatings therefore show promising robustness under physiological conditions. IPN coatings prevent four types of surgically and physiologically associated damage. In the future, additional studies will examine characterization of IPN cohesive strength to a variety of substrates.

### Biological properties of IPN hydrogel coatings

After demonstrating that the IPN hydrogel coatings were damage resistant, we characterized the thromboresistance and bioactivity of the coatings. It is essential that the coatings prevent platelet adhesion and activation for acute thromboresistance and can incorporate bioactive proteins to promote endothelialization for sustained thromboresistance. As an initial assessment that the IPN formulation retained the antifouling nature of PEG-based hydrogels, both protein adsorption and static platelet attachment were characterized. Then, functionalized collagen was added into the IPN hydrogel coating and endothelial cell adhesion was assessed.

**Fig. 5:**
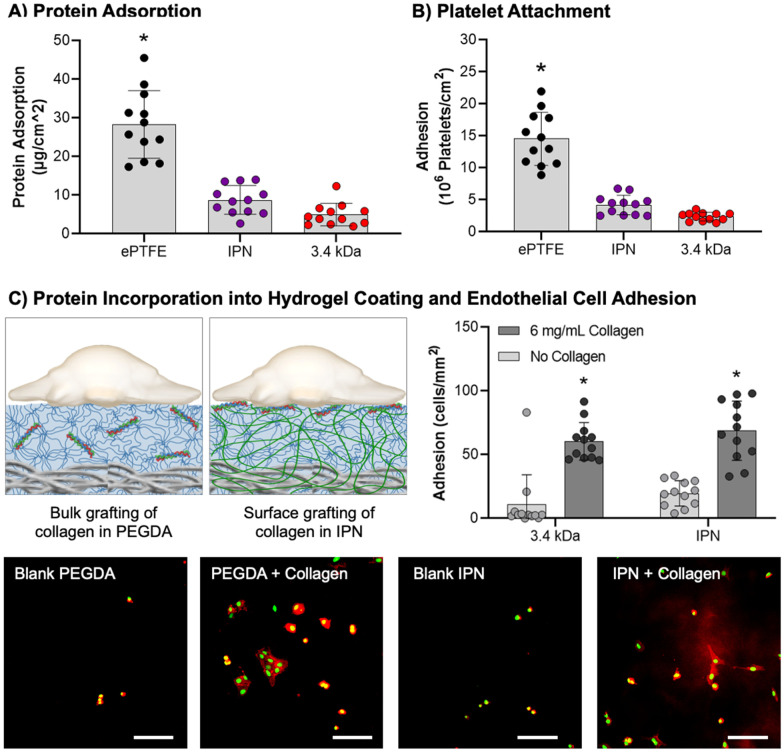
Biological interactions of IPN hydrogel coatings. (A) Protein adsorption to material surfaces (*n* = 12). (B) Static platelet attachment to material surfaces (*n* = 12). (C) Schematic depiction of protein incorporation in PEGDA and IPN hydrogel coatings with resulting HUVEC adhesion to hydrogels with or without functionalized collagen, scale bar = 100 μm (*n* = 12). The * represents a difference between groups (*p* < 0.05) in ANOVA with Tukey's multiple comparison test. Data for all comparisons represents average ± standard mean deviation.

Protein adsorption is the first step of the coagulation cascade that leads to acute thrombosis. Thus, protein adsorption was assessed as an initial measure of thromboresistance (Fig. 5(A)). IPN and 3.4 kDa hydrogel coatings had the lowest protein adsorption at 8.8 ± 3.7 and 5.0 ± 2.9 μg cm^−2^, respectively, significantly lower than the ePTFE clinical control (*p* < 0.0001, *n* = 12). Attachment to the different hydrogel coating compositions was not significantly different. Previous studies of PEG hydrogel coatings have demonstrated minimal protein adsorption.^11^ Surfaces that are charge-neutral and have a strongly absorbed surface water layer are able to reduce protein adsorption *via* competitive hydrogen bonding interactions with water molecules.^91^ The strong intermolecular hydrogen bonding of pNAGA hydrogels was suspect to increase protein adsorption to the IPN hydrogel coatings. Recently, Wang and coworkers demonstrated a zwitterionic pNAGA copolymer with carboxybetaine acrylamide that showed reduced protein adsorption over pNAGA networks alone.^108^ Zwitterionic polymers are known to have non-fouling properties and mechanisms similar to PEG.^91^ This work indicates strategies that increase the hydrophilicity of pNAGA networks such as the IPN approach implemented here can limit hydrogel biofouling.

Previous iterations of PEG-based hydrogel coatings developed in our lab demonstrated thromboresistance.^7,8^ To assess retention of these properties in the IPN hydrogel, static platelet attachment was characterized (Fig. 5(B)). All samples were coated with an FBS soak to better mimic physiological conditions. Platelet attachment was highest for the ePTFE graft (10 ± 0.92 × 10^6^ platelets per cm^2^). PEGDA 3.4 kDa and IPN hydrogel coatings had significantly lower platelet attachment as compared to the clinical control (*p* < 0.0001, *n* = 12). There was not a significant difference between the PEGDA 3.4 kDa and IPN hydrogel coatings, indicating minimal impact of hydrogel chemistry on platelet adhesion. Further characterization of platelet activation and hemolysis must be conducted to determine the extent of innate thromboresistant properties of these materials. However, the static platelet attachment results here are satisfactory to demonstrate IPN hydrogel coatings are not significantly more thrombogenic than PEGDA 3.4 kDa coatings.

Sustained thromboresistance *via* the recruitment of an endothelium post-implantation is essential for sustained thromboresistance. The bioactivity of IPN hydrogel coatings was investigated by incorporating functionalized collagen at the surface of these networks and assessing the ability of endothelial cells to differentially adhere to these bioactive substrates (Fig. 5(C)). HUVEC attachment studies demonstrated a significant increase in HUVEC adhesion to both 3.4 kDa and IPN samples with incorporated collagen as opposed to blanks (*p* < 0.0001, *n* = 12, Fig. 5(C)). HUVEC attachment was not significantly different between the 3.4 kDa and IPN hydrogel samples with 0.1× collagen (60 ± 15 and 69 ± 23 cells per mm^2^) respectively. Further, endothelial cell spreading was visualized on both 3.4 kDa and IPN hydrogels after 3 hours. These results indicate that the surface properties of bioactive IPN hydrogel coatings are similar enough to those of PEGDA 3.4 kDa coatings to mimic their bioactive properties. Future endothelial cell studies are needed to characterize cell migration rates and hemostatic phenotypes following confluence. These results combined with resistance to protein adsorption and platelet attachment are promising indicators that the damage-resistant IPN coatings retained the thromboresistance of other PEG-based hydrogels.

## Conclusions

The development of a durable and bioactive hydrogel coating for cardiovascular devices would have significant impact on clinical outcomes. This work sought to eliminate the damage mechanisms that lead to hydrogel coating failure during surgical implantation procedures while maintaining the desirable thromboresistance and bioactivity of the previous hydrogel formulation. The IPN hydrogel coating was designed using a primary network of a biostable PEG-based macromer supported by a secondary network formed from the bidentate hydrogen bonding monomer, NAGA. This IPN matched the modulus of previous bioactive hydrogels and greatly improved the mechanical properties and damage resistance of the hydrogel coating. Further, the IPN coating showed resistance to protein adsorption, platelet attachment, and successfully incorporated bioactive proteins to support endothelial cell adhesion and spreading. The methods for forming conformable and highly tunable coatings conformal to material substrates presented in this work can be adapted to other macromers and porous substrates in a straightforward manner. This work adds important contribution to the current rise in innovation of robust hydrogel coatings for biomedical devices.

## Conflicts of interest

There are no conflicts to declare.

## Supplementary Material

TB-011-D2TB02825E-s001
