# Supplementary material for: Interpenetrating network design of bioactive hydrogel coatings with enhanced damage resistance
Source: J Mater Chem B. 2023 Feb 20;11(24):5416–28. doi: 10.1039/d2tb02825e (PMC10682960; doi:10.1039/d2tb02825e)
Supplement: TB-011-D2TB02825E-s001 [file TB-011-D2TB02825E-s001.pdf]

Electronic Supplementary Material (ESI) for Journal of Materials Chemistry B.
This journal is © The Royal Society of Chemistry 2023

# Interpenetrating Network Design of Bioactive Hydrogel Coatings with Enhanced Damage Resistance

*Megan Wancura[a], Abbey Nkansah[b], Malgorzata Chwatko[b⸸], Andrew Robinson[b], Ashauntee Fairley[b], Elizabeth Cosgriff-Hernandez[b*]*

a. Department of Chemistry, The University of Texas at Austin, Austin TX 78712, USA

b. Department of Biomedical Engineering, The University of Texas at Austin, Austin TX 78712, USA

⸸ Present location: Department of Chemical and Materials Engineering; University of Kentucky, Lexington, KY 40506, USA

## Supplementary Information

*Synthesis of PEGDA*

Poly(ethylene glycol) diacrylate (PEGDA) was synthesized as described by Browning et al. with minor modifications.[7] Briefly, triethylamine (2 equiv.) was added dropwise to a solution of PEG (3.4 kDa or 10 kDa, 0.1 mmol/mL; 20 kDa, 15wt%; 1 equiv.) in anhydrous dichloromethane under nitrogen atmosphere. Acryloyl chloride (4 equiv.) was added dropwise (1 drop every 4-5 s), and the reaction was stirred for 24 h. For fabrication of PEGDA 20 kDa, the reaction was allowed to stir for 48 h with additional acryloyl chloride (2 mole equivalents) added dropwise after 24 h. The reaction was then washed with potassium bicarbonate (8 equiv.) and dried with anhydrous sodium sulfate. PEGDA was precipitated in cold diethyl ether, filtered, and dried at room temperature overnight followed by vacuum drying. The degree of acrylation of the product was determined using proton nuclear magnetic resonance ($^1$H NMR) spectroscopy. Spectra were recorded on a Varian MR-400 400 MHz spectrometer and analyzed using a TMS/solvent signal as an internal reference. Polymers with percentage conversions of hydroxyl to acrylate end groups over 80% were used in this work. $^1$H-NMR ($CDCl_3$): 3.6 ppm (m, $–OCH_2CH_2–$), 4.3 ppm (t, $–CH_2OCO–$), 6.1 ppm (dd, $–CH=CH_2$), 5.8 and 6.4 ppm (dd, $–CH=CH_2$).

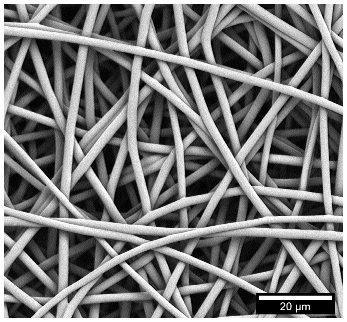


**Figure S1**: Scanning electron micrograph of electrospun Bionate® segmented polyurethane mesh.

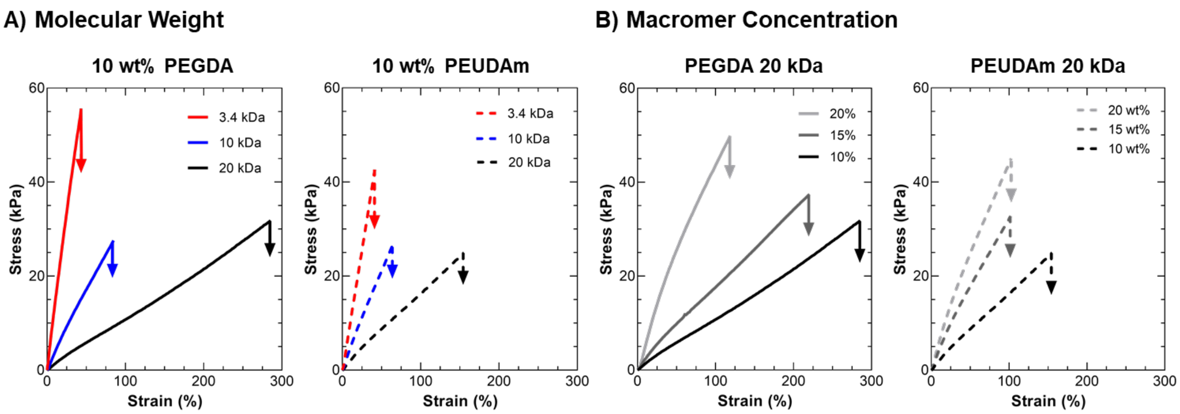


**Figure S2**: Tensile and fracture properties of PEGDA and PEUDAm hydrogel networks at multiple molecular weights (3.4, 10, and 20 kDa, 10 wt%) and concentrations (10, 15, and 20 wt%, 20 kDa). Representative tensile curves of PEGDA and PEUDAm. Impact of molecular weight on PEGDA (**A**) and PEUDAm (**B**). Impact of concentration on PEGDA (**C**) and PEUDAm (**D**).

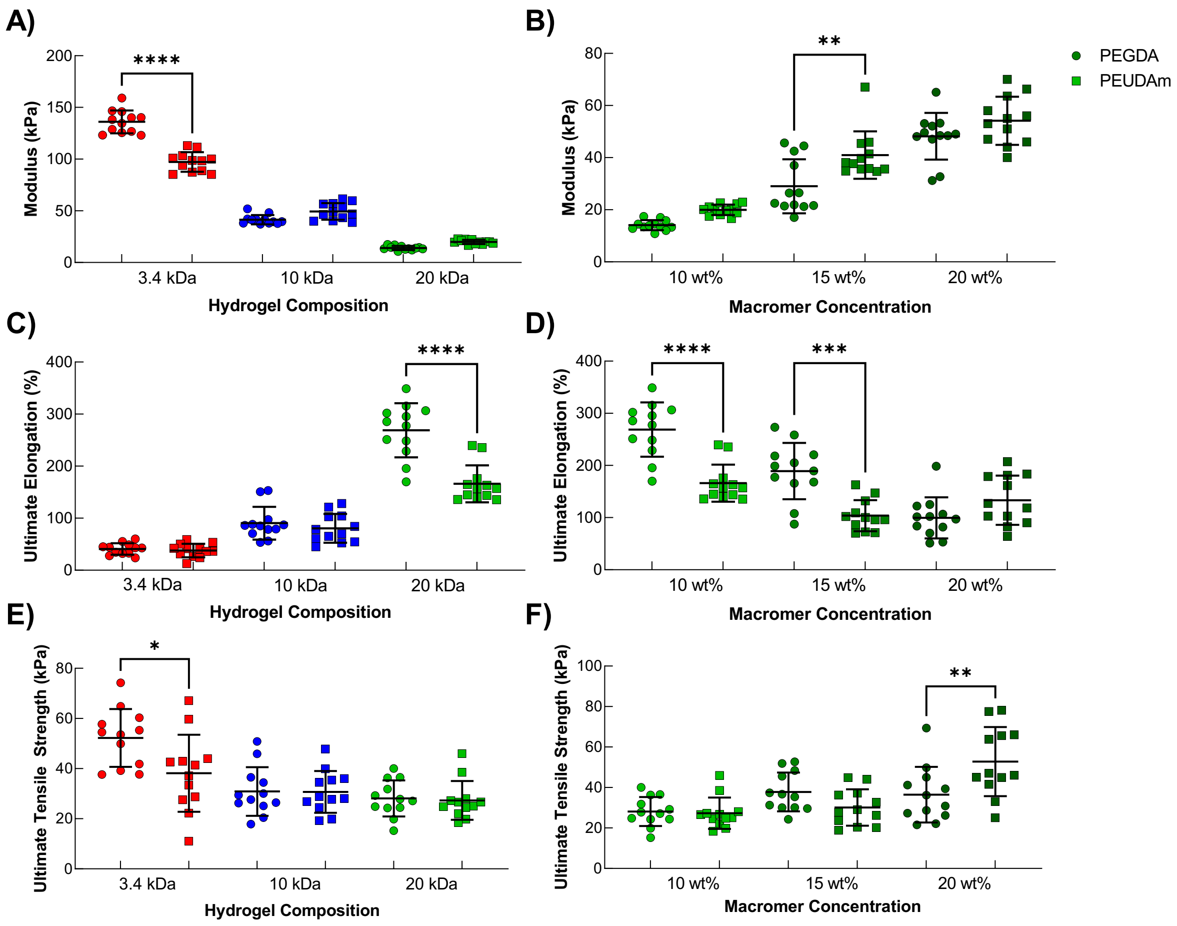


**Figure S3:** Tensile mechanical data comparisons for PEGDA and PEUDAm hydrogel networks as an effect of macromer molecular weight and concentration. **A**) Tensile modulus as an effect of

molecular weight. **B**) tensile modulus as an effect of macromer concentration. **C**) Ultimate elongation as an effect of molecular weight. **D**) ultimate elongation as an effect of macromer concentration. **E**) Ultimate tensile strength as an effect of molecular weight. **F**) Ultimate tensile strength as an effect of macromer concentration. * represents $p < 0.05$; ** represents $p < 0.01$; *** represents $p < 0.001$, and **** represents $p < 0.0001$. All error bars represent standard deviation.

**Table S1:** First Network Effects on Mechanical Properties

| Macromer | Molecular Weight | Concentration (wt%) | Swelling Ratio (Q) | Gel Fraction (%) | Tensile Modulus (kPa) | Ultimate Elongation (%) | Ultimate Tensile Strength (kPa) |
|---|---|---|---|---|---|---|---|
| PEGDA | 3.4 | 10 | 11 ± 0.21 | 97.0 ± 4.19 | 136 ± 11.1 | 40 ± 11 | 52 ± 12 |
| | 10 | 10 | 21 ± 0.35 | 97.4 ± 2.01 | 41.4 ± 4.47 | 90 ± 32 | 31 ± 10 |
| | 20 | 10 | 30 ± 0.99 | 97.4 ± 1.27 | 14.1 ± 1.93 | 270 ± 52 | 28 ± 7.2 |
| | 20 | 15 | 23 ± 1.5 | 95.8 ± 2.47 | 29.0 ± 10.4 | 190 ± 54 | 38 ± 9.6 |
| | 20 | 20 | 17 ± 0.12 | 96.7 ± 3.82 | 48.2 ± 8.96 | 99 ± 39 | 36 ± 14 |
| PEUDAm | 3.4 | 10 | 11 ± 0.26 | 92.1 ± 5.22 | 97.2 ± 9.48 | 38 ± 13 | 38 ± 15 |
| | 10 | 10 | 18 ± 0.31 | 97.4 ± 1.27 | 48.4 ± 8.07 | 81 ± 28 | 31 ± 8.3 |
| | 20 | 10 | 29 ± 0.33 | 95.7 ± 1.73 | 20.0 ± 2.02 | 170 ± 36 | 27 ± 7.7 |
| | 20 | 15 | 21 ± 0.23 | 97.4 ± 0.488 | 41.0 ± 9.09 | 100 ± 30 | 30 ± 9.0 |
| | 20 | 20 | 17 ± 0.12 | 98.6 ± 0.356 | 54.2 ± 9.24 | 130 ± 47 | 53 ± 17 |

Tensile properties PEUDAm single networks were determined at multiple molecular weights and concentrations relative to PEGDA networks. Increasing molecular weight resulted in significantly increased ultimate elongation, decreased ultimate tensile strength, and decreased modulus for PEGDA networks, **Figure S2**, ($p < 0.0001$, $n = 12$). For PEUDAm networks, increased molecular weight did not significantly affect ultimate tensile strength. Increasing macromer concentration for 20 kDa PEGDA networks resulted in significantly decreased ultimate elongation and increased modulus for PEGDA networks ($p < 0.0001$, $n = 12$); whereas, no statistical difference in ultimate tensile strength was observed. Increased macromer concentration for 20 kDa PEUDAm networks resulted in significantly decreased ultimate elongations, increased modulus, and increased ultimate tensile strengths ($p < 0.005$, $n = 12$).

Comparison of PEGDA and PEUDAm networks proved most differences to be insignificant, indicating high structural similarity between the hydrogels. A clear difference was the lower modulus of PEUDAm 3.4 kDa relative to PEGDA 3.4 kDa (97.2 ± 9.48 vs. 136 ± 11.1 kPa, $p < 0001$, $n = 12$, **Figure S2**). Ultimate elongations of these networks were not significantly different (38 ± 13 vs 40 ± 11%, $n = 12$), nor were swelling ratios (11 ± 0.21 vs. 11 ± 0.26, $n = 12$, **Table S1**). Additionally, PEUDAm 20 kDa had lower ultimate elongations than PEGDA 20 kDa at 10 and 15 wt% (170 ± 36 vs. 270 ± 52% at 10 wt%, $p < 0.0001$; 104 ± 30 vs. 190 ± 54% at 15 wt%, $p < 0.001$, $n = 12$, **Figure S2**). Secondary interactions between the urethane bonds near the acrylamide reactive groups are possible, though sterically hindered. However, if these interactions strongly affected network structure, it is likely clearer trends would have arisen in characterization. Due to the higher crosslinking density of the 3.4 kDa networks, it is possible that secondary interactions more strongly affect these hydrogels than higher molecular weights, resulting in modulus effects. Differences in 20 kDa networks could be caused by the dilute number of reactive groups in these systems leading to higher heterogeneity.[107] Further characterization of network structure and likelihood of secondary interactions at crosslinking points would be necessary to determine any impact of the urethane motifs.

**Table S2:** Interpenetrating Network Effects on Mechanical Properties

| Macromer | [NAGA] (wt%) | Swelling Ratio (Q) | Gel Fraction (%) | Tensile Modulus (kPa) | Ultimate Elongation (%) | Ultimate Tensile Strength (kPa) | Fracture Elongation (%) | Maximum Force at Fracture (N) | Fracture Energy (J/mm^2) |
|---|---|---|---|---|---|---|---|---|---|
| PEGDA 20 kDa | - | 30 ± 0.99 | 97.4 ± 1.27 | 14.1 ± 1.93 | 270 ± 52 | 28 ± 7.2 | - | - | - |
| | 20 | 9.4 ± 0.45 | 95.8 ± 0.766 | 54.0 ± 10.8 | 150 ± 52 | 64 ± 24 | - | - | - |
| PEUDAm 20 kDa | - | 29 ± 0.33 | 95.7 ± 1.73 | 20.0 ± 2.02 | 170 ± 36 | 27 ± 7.7 | 26 ± 6 | 0.07 ± 0.04 | 0.03 ± 0.02 |
| | 10 | 10 ± 0.70 | 87.7 ± 2.92 | 24.7 ± 2.71 | 130 ± 45 | 32 ± 12 | - | - | - |
| | 15 | 8.2 ± 1.2 | 89.5 ± 3.24 | 62.4 ± 4.25 | 140 ± 62 | 76 ± 33 | - | - | - |
| | 20 | 6.1 ± 1.3 | 93.5 ± 4.80 | 118 ± 24.8 | 280 ± 76 | 240 ± 71 | 34 ± 5 | 0.34 ± 0.09 | 0.22 ± 0.08 |
| pNAGA | 20 | 3.4 ± 0.14 | 94.7 ± 1.70 | 207 ± 17.0 | 280 ± 84 | 450 ± 92 | 76 ± 12 | 0.95 ± 0.08 | 1.63 ± 0.38 |

For all mechanical properties, IPNs with a PEUDAm vs. PEGDA first network had increased properties (modulus: PEGDA: 57.6 ± 13.6 kPa vs. PEUDAm: 118 ± 24.8 kPa; ultimate elongation: PEGDA: 160 ± 43% vs. PEUDAm: 280 ± 76%; ultimate tensile strength: PEGDA: 81 ± 33 kPa vs. PEUDAm: 240 ± 71 kPa, $p < 0.001$, **Figure S5**). Second network incorporation at 20 wt% NAGA decreased ultimate elongation in PEGDA networks and increased ultimate elongation in PEUDAm networks (PEGDA SN: 270 ± 52% vs. IPN: 160 ± 43%, $p < 0.001$; PEUDAm SN: 170 ± 36% vs. IPN: 280 ± 76%, $p = 0.0003$; $n = 12$). Ultimate tensile strength was significantly increased only for PEUDAm networks (PEGDA SN: 28 ± 7.2 kPa vs. IPN: 81 ± 33 kPa; PEUDAm SN: 27 ± 7.7 vs. IPN: 240 ± 71 kPa, $p < 0.0001$; $n = 12$). Differences between PEGDA and PEUDAm networks may be attributable to the urethane motifs in the PEUDAm crosslinking points.

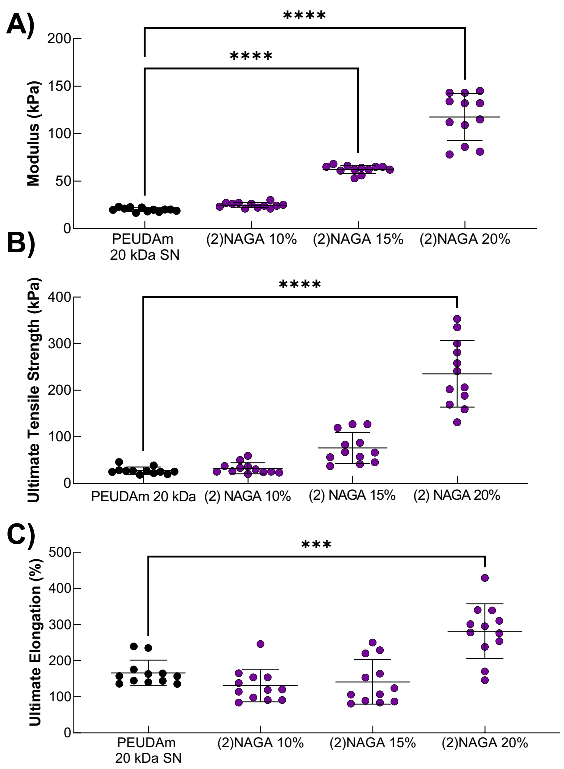


**Figure S4:** Relative tensile properties of PEUDAm IPN networks with varying concentrations of NAGA. **A**) Young's modulus, **B**) ultimate tensile strength, and **C**) ultimate elongation. All

comparisons represent an n = 12 and error bars represent standard deviation. *** represents $p < 0.001$, and **** represents $p < 0.0001$.

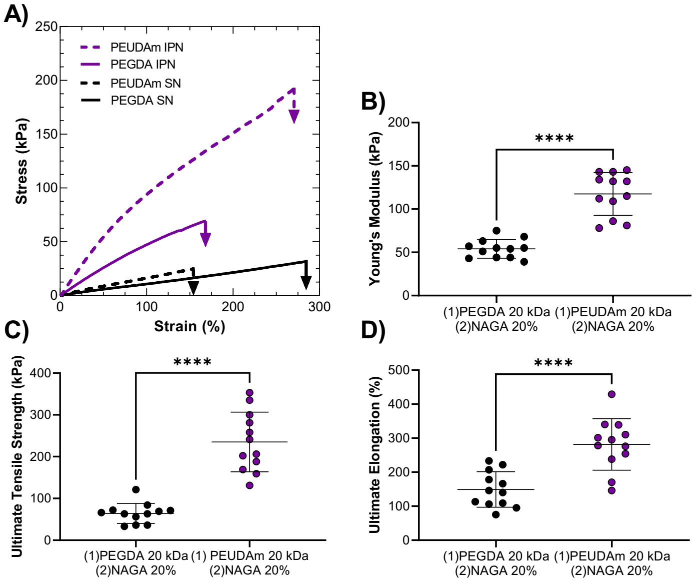


**Figure S5:** Relative tensile properties of PEGDA and PEUDAm IPN networks. **A**) Representative curves. Effect of PEGDA vs. PEUDAm on IPN network **B**) Young's modulus, **C**) ultimate tensile strength, and **D**) ultimate elongation. All comparisons represent an n = 12 and error bars represent standard deviation. *** represents $p < 0.001$, and **** represents $p < 0.0001$.

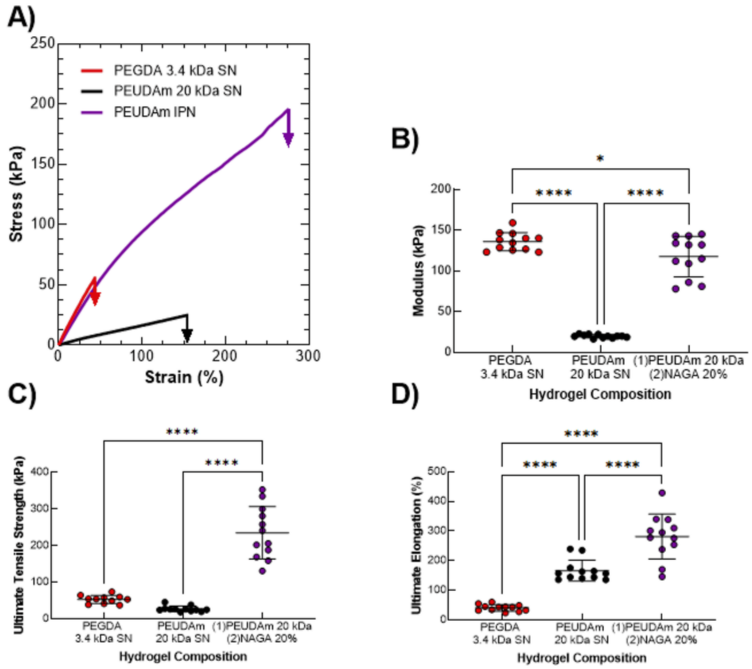


**Figure S6:** Tensile properties of PEUDAm IPNs relative to PEGDA 3.4 kDa networks. **A**) Representative curves. **B**) Young's modulus. **C**) Ultimate tensile strength. **D**) Ultimate elongation.

All comparisons represent an n = 12 and error bars represent standard deviation. * represents $p < 0.05$, and **** represents $p < 0.0001$.

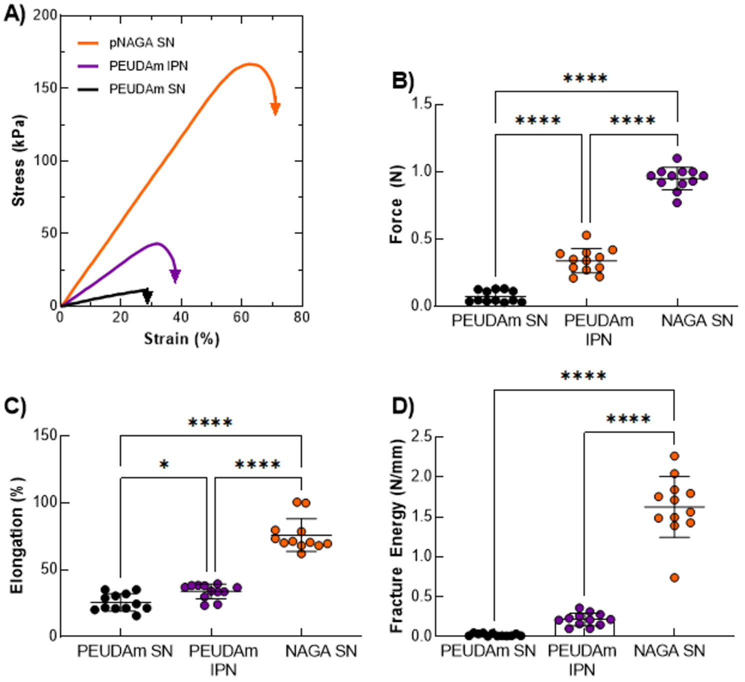


**Figure S7:** Fracture properties of PEUDAm IPN networks. **A**) Representative curves. Effect of IPN on **B**) maximum force at break, **C**) elongation at break under fracture, and **D**) fracture energy. All comparisons represent an n = 12 and error bars represent standard deviation. * represents $p < 0.05$, and **** represents $p < 0.0001$.

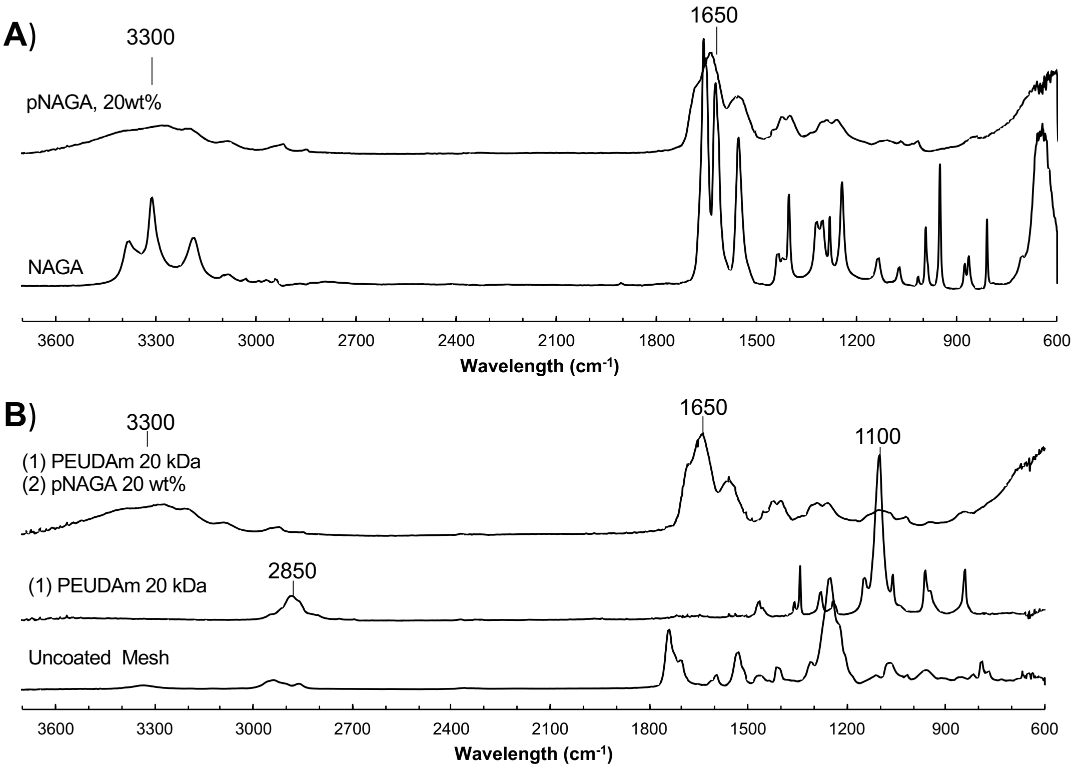


**Figure S8**: FTIR characterization of bulk hydrogels and hydrogel coatings. **A**) pNAGA dry bulk hydrogel and NAGA monomer. **B**) IPN hydrogel coating compared to uncoated mesh and first network PEUDAm.

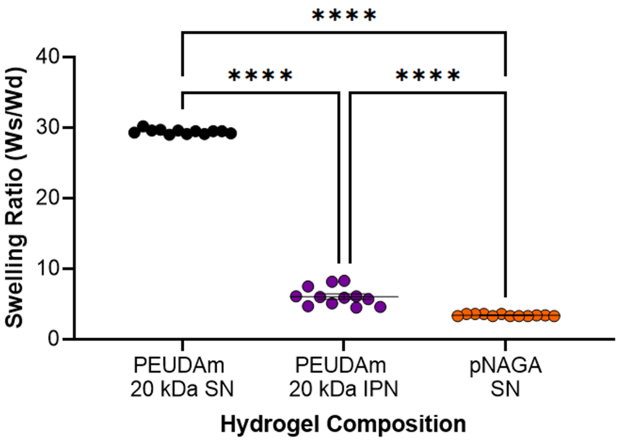


**Figure S9:** Swelling ratios of PEUDAm 20 kDa IPNs relative to component single networks. **** represents $p < 0.0001$.

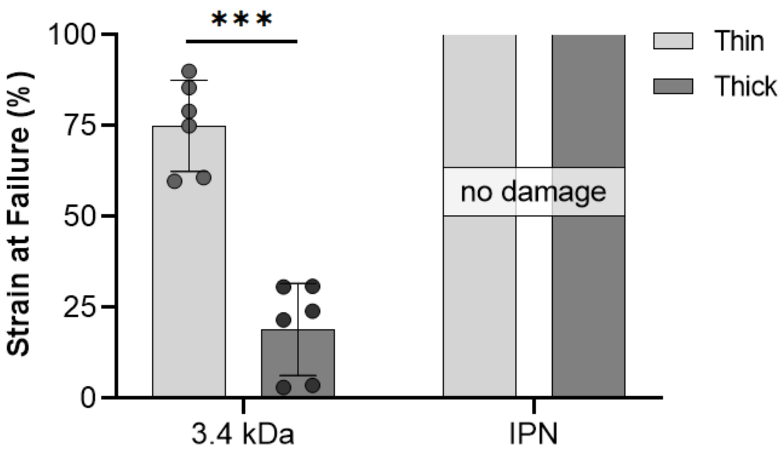


**Figure S10:** Stretch damage of hydrogel composites as an effect of coating thickness. *** represents $p < 0.0001$.
